# MDM2 mediates p73 ubiquitination: a new molecular mechanism for suppression of p73 function

**DOI:** 10.18632/oncotarget.4086

**Published:** 2015-05-26

**Authors:** Hong Wu, Roger P. Leng

**Affiliations:** ^1^ Department of Laboratory Medicine and Pathology, University of Alberta, Edmonton, Alberta, Canada

**Keywords:** ubiquitin E3 ligase, ubiquitination, tumor suppressor, oncogene, protein degradation

## Abstract

The protein p73, a homologue of the tumor suppressor protein p53, is capable of inducing apoptosis and cell cycle arrest. MDM2 is transcriptionally activated by p73 and represses the functions of p73, including p73-dependent transactivation and growth suppression. However, the molecular mechanism of this repression is unknown. In this study, we show that MDM2 mediates p73 ubiquitination. MDM2 mainly utilizes K11, K29 and K63-linked chains to mediate p73 ubiquitination *in vivo* and *in vitro*. However, MDM2 is unable to promote p73 degradation in most tested cell lines. Surprisingly, we observe that overexpression of Mdm2 promotes p73 degradation mainly through Itch in Mdm2-null MEFs. We further find that Itch interacts with the transfected Mdm2 in Mdm2-null cells. Moreover, our findings reveal that the E3 ligase activity of MDM2 is required to repress p73-dependent apoptosis and cell cycle arrest but not p73-dependent transcriptional activity. Furthermore, the data suggest a link between p73 ubiquitination/MDM2 E3 ligase activity and p73 biological functions.

## INTRODUCTION

The protein p73, which is a homologue of p53, is capable of inducing apoptosis or cell cycle arrest [1–4]. Although p73 has apoptotic activity, it is rarely mutated in human tumors, and p73 deficient mice do not have an increase in tumor incidence [1]. The literature describing the tumor-suppressor function of p73 is complex and controversial, and the role of p73 has been a matter of debate. However, a number of studies have recently shown that p73 expression is lost or reduced in certain human tumors (loss of heterozygosity, allele silencing, etc.), indicating that p73 has tumor-suppressing activities [5–7]. Mice that are heterozygous for p73 develop spontaneous tumors; the loss of p73 can cooperate with p53 in tumor suppression; and tandem mutation of p73 and p53 leads to a more aggressive tumor phenotype. These findings indicate that p73 plays an important role in tumor suppression in mice [8]. Itch was identified as an E3 ligase for p73 [9]; however, multiple pathways may be involved in the control of p73 functions.

Mdm2 was originally identified as an amplified gene in a spontaneously transformed derivative of mouse BALB/c cells [10]. Mdm2 (known as MDM2 or Hdm2 in humans) interacts with the p53 transactivation domain [11, 12] and promotes p53 degradation via the ubiquitinproteasome pathway [13–15]. The deletion of Mdm2 in mice results in an embryonic lethal phenotype, but lethality can be prevented by deletion of the p53 gene, indicating the importance of the negative regulatory function of Mdm2 on p53 during development [16, 17]. Interestingly, MDM2 is transcriptionally activated by p73 and represses many of its functions, such as p73-dependent transactivation and growth suppression [18, 19]. However, the molecular mechanisms of this repression are unknown.

In this study, we observe that MDM2 promotes p73 ubiquitination. We show that MDM2 is required to mediate p73 ubiquitination *in vivo*. MDM2 is unable to promote p73 degradation in most tested cell lines. However, our findings reveal that overexpression of Mdm2 promotes p73 degradation in Mdm2-null MEFs, and is dependent on Itch. Depletion of MDM2 by siRNA activates p73-dependent transactivation and apoptosis. In addition, the RING domain of MDM2 is essential for MDM2-mediated p73 ubiquitination.

## RESULTS

### Overexpression of MDM2 mediates the ubiquitination of p73 *in vivo*

**Figure 1:**
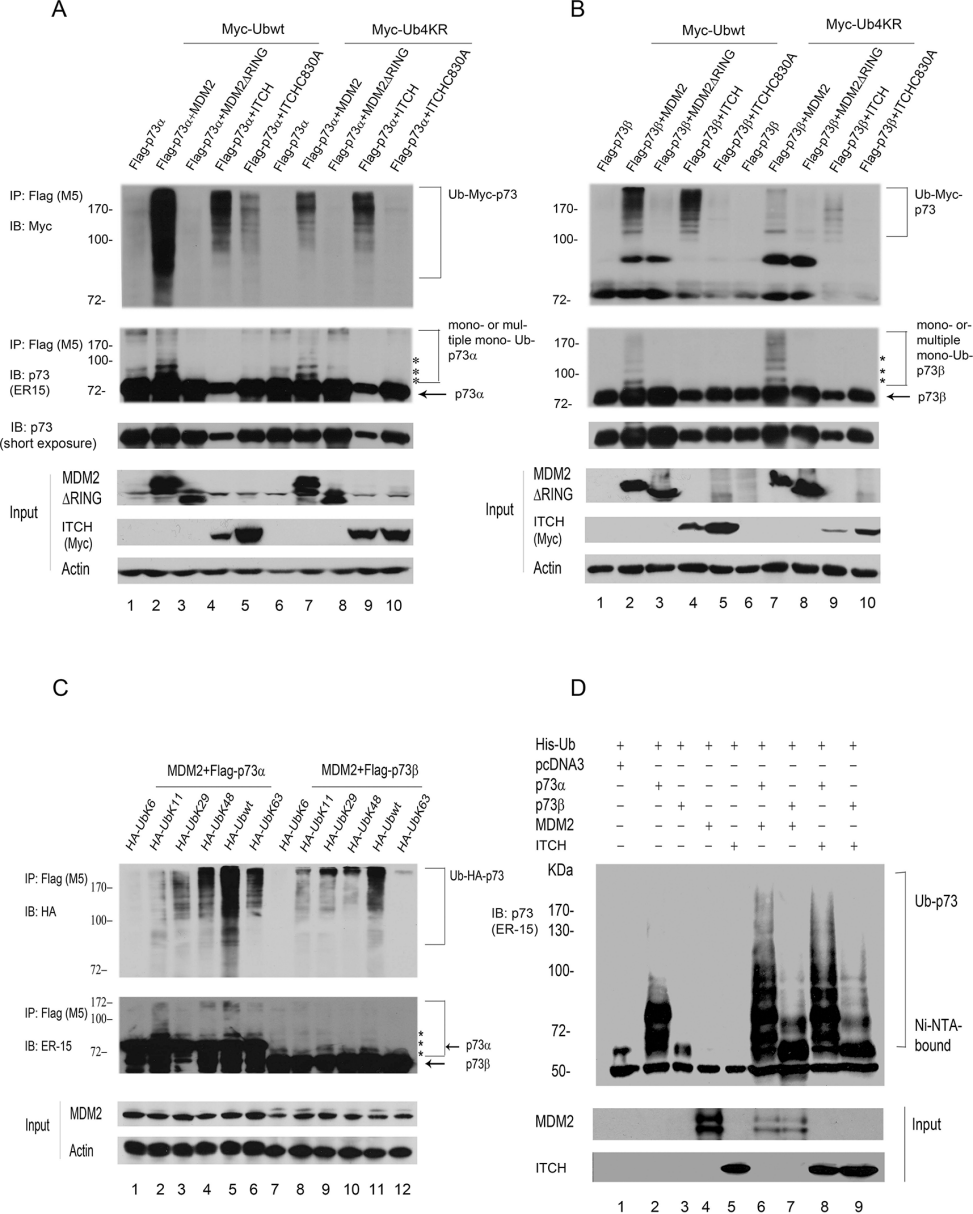
MDM2 promotes p73 ubiquitination *in vivo* **A.** Saos-2 cells were cotransfected with plasmids expressing Flag-p73α and pCMV-Bam-MDM2, MDM2ΔRING, ITCH, or ITCH mutant (C830A) as well as Myc-Ubwt or Myc-Ub4KR as indicated. p73 was immunoprecipitated with a Flag-specific antibody (M5) and analyzed by immunoblotting with a Myc-specific antibody (upper image) and ER-15 for p73 (second image). Direct western blots for MDM2 and ITCH are shown in the lower panels. **B.** The same procedure as (A) was used, except that Saos-2 cells were cotransfected with a Flag-p73β-expressing plasmid instead of Flag-p73α. **C.** The same procedure as (A) was used, except that Saos-2 cells were cotransfected with plasmids encoding p73α or p73β and MDM2 along with a number of ubiquitin mutants as indicated. Asterisk (*) indicates the migration position of p73-Ub conjugates. **D.** Saos-2 cells were transfected with plasmids expressing His-ubiquitin, p73α or p73β, and MDM2 or ITCH. His-ubiquitinated proteins were isolated from denatured whole extract extracts, and analyzed by western blot with a p73 specific antibody (ER-15). Direct western blots for MDM2 and ITCH are shown in the lower panels.

MDM2 is a ubiquitin E3 ligase for p53 and promotes p53 degradation [15, 23, 24]. MDM2 has been reported to bind p73 and suppress its function without inducing degradation [18, 19]. We investigated whether ectopic expression of MDM2 promotes p73 ubiquitination *in vivo*. Accordingly, p53-negative Saos-2 cells were transfected with expression plasmids encoding p73α or p73β alone or with MDM2, MDM2ΔRING (in which the RING domain has been deleted), ITCH, or an ITCH HECT mutant. In addition, cells were cotransfected with Myc-tagged wild-type ubiquitin (Ubwt) or Myc-tagged Ub4KR as indicated. Ub4KR is a partial polyubiquitination-defective mutant (in which Lys11, Lys29, Lys48, and Lys 63 were each replaced by arginine, kindly provided by Dr. Y. Yarden). *In vivo* ubiquitination assays were performed under denaturing conditions [20–22]. Immunoprecipitation of p73 with a Flag-specific antibody (M5) was followed by immunoblotting with a Myc-specific antibody to detect ubiquitinated p73 (Figure 1A, 1B, upper image) or a p73-specific antibody (ER-15) to detect all p73 (Figure 1A, 1B, lower image). Immunoprecipitated p73 (or proteins associated with p73) were heavily ubiquitinated in the presence of MDM2 and wild-type ubiquitin (Ubwt) (Figure 1A, 1B, upper image). However, MDM2ΔRING was unable to promote p73 ubiquitination, indicating that the RING domain of MDM2 is essential for its E3 ligase activity (Figure 1A, 1B). As shown in Figure 1A, and 1B, Ub4KR results in significantly reduced MDM2-mediated p73 ubiquitination compared to Ubwt, suggesting that MDM2-mediated p73 ubiquitination occurs predominantly on Lys11, and/or Lys29, and/or Lys48, and/or Lys63 of ubiquitin *in vivo*. Saos-2 cells coexpressing Flag-p73 and MDM2 along with the Myc-tagged Ub4KR mutant recapitulate the Ubwt pattern; however, the levels of p73 ubiquitination are markedly reduced (Figure 1A, 1B). Consistent with previous reports [18, 19], MDM2 is unable to decrease the levels of p73α and p73β proteins in Saos-2 cells (third images, Figure 1A, 1B). These data indicate that MDM2 promotes predominantly multiple or poly-ubiquitination of p73 *in vivo*. These findings support the hypothesis that MDM2 plays an important role in promoting p73 ubiquitination. Similar results were obtained using H1299 cells (Supplementary Figure S1). It was reported that Itch is an E3 ligase for p73 that negatively regulates p73 stability [9]. We overexpressed MDM2 and ITCH/AIP4 (the human homolog of mouse Itch) in Saos-2 cells using plasmids expressing Ubwt or the Ub4KR mutant to mediate ubiquitination of p73. Notably, we detected weak p73 ubiquitination in the ITCHC830A mutant, suggesting that this point mutant of Itch may have weak E3 ligase activity (Figure 1A, 1B). To identify the lysine residue(s) of ubiquitin (Ub) required for p73 ubiquitination by MDM2 *in vivo*, we examined a number of Ub mutants. The mutants each contained one lysine, with the remaining five lysine residues (K6, K11, K29, K48, and K63) mutated to arginine. p73α or p73β and HA-tagged ubiquitin or various HA-tagged ubiquitin mutants were coexpressed with MDM2 in Saos-2 cells. As shown in Figure 1C, our finding suggested that MDM2 may preferentially utilize multiple Lys residues of ubiquitin to promote p73 ubiquitination *in vivo*. The p73 immunoblots revealed that MDM2 promotes polyubiquitination of p73 *in vivo* (Figure 1C, lower image). To provide direct evidence that the modified p73 species corresponds to ubiquitin conjugation, we coexpressed His-tagged ubiquitin and p73α or p73β with or without MDM2 or ITCH in Saos-2 cells and isolated His-ubiquitin conjugated proteins under denaturing conditions. Ubiquitin conjugation was detected in the presence of MDM2, as did the coexpression of p73 and ITCH (Figure 1D). Taken together, these data demonstrate that MDM2 promotes p73 ubiquitination *in vivo*.

### MDM2 is required for p73 ubiquitination *in vivo*

**Figure 2:**
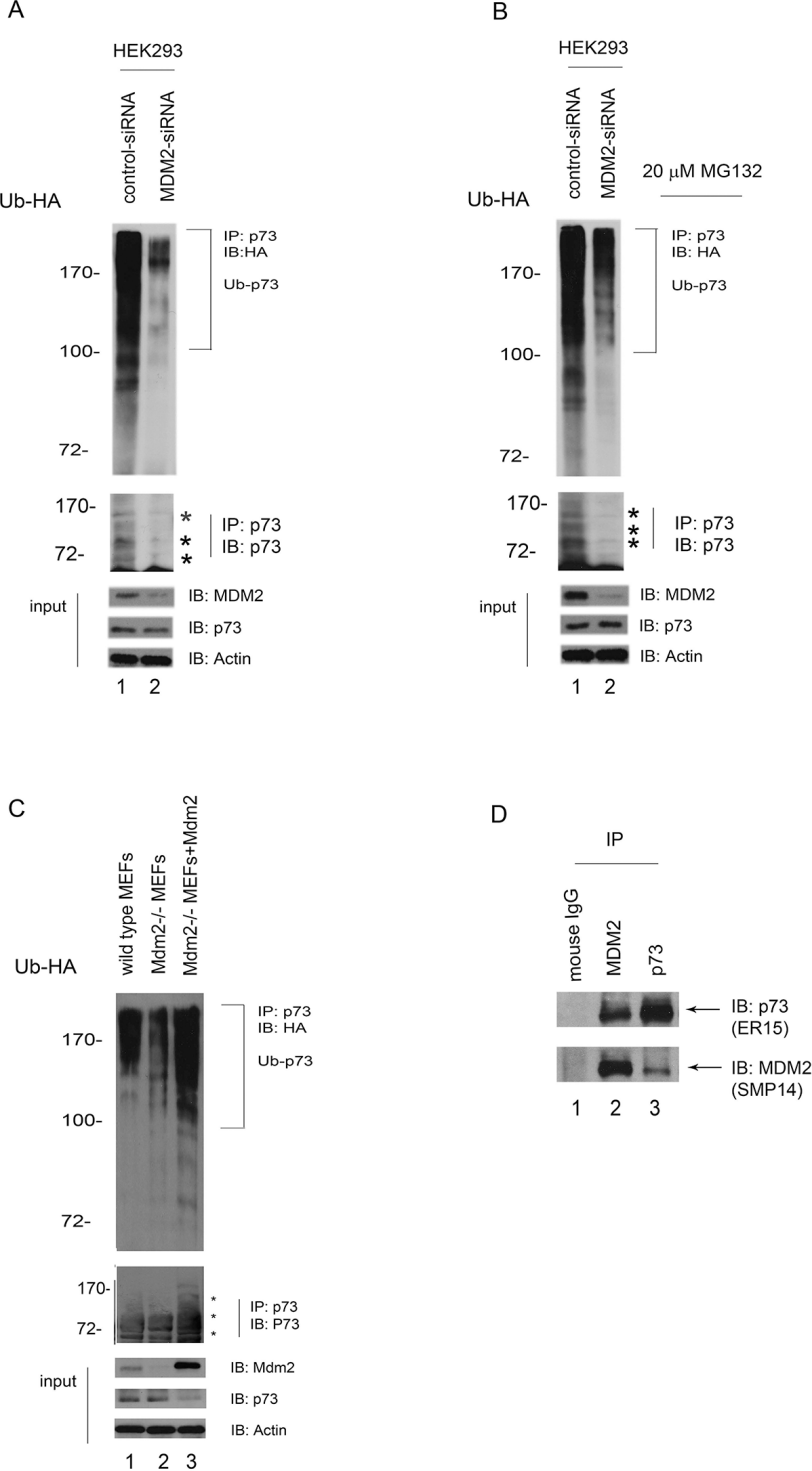
MDM2 is required for p73 ubiquitination *in vivo* **A.** HEK293 cells were transfected with MDM2-siRNA or control-siRNA constructs. Forty hours later, the cells were further transfected with a plasmid expressing HA-Ub. Lysates were immunoprecipitated with a p73specific antibody (ER-15) and analyzed by western blotting with an HA-specific antibody to detect ubiquitinated p73. The western blots for p73, MDM2, and actin are shown in the lower panel. **B.** The same procedure as (A) was used, except that after the second transfection with the HA-Ub expression plasmid, the cells were treated with the proteasome inhibitor MG132 (20 μM) 6 hr prior to harvest. **C.** Similar to (A) except that the cell extracts were obtained from the wild-type mouse embryonic fibroblasts (MEFs) and Mdm2 null MEFs. In addition, Mdm2 expression plasmid was reintroduced into Mdm2 null MEFs (lane 3). **D.** HEK293 cells were immunoprecipitated with anti-p73 (ER-15) or anti-MDM2 (SMP14) as indicated, and immunoblotted with antip73 (upper image, ER-15) and anti-MDM2 antibodies (lower image).

To determine whether MDM2 functions as an E3 ligase for p73 *in vivo*, HEK293 cells were transfected with control-siRNA or MDM2-siRNA constructs. After 40 hours, the cells were transfected with an HA-tagged ubiquitin expression plasmid and immunoprecipitated with a p73specific antibody (ER-15) under denaturing conditions. Ubiquitination of p73 was markedly decreased upon MDM2-siRNA treatment compared with control-siRNA treatment (Figure 2A). Stronger ubiquitination of p73 was observed when cells were treated with the proteasome inhibitor MG132, suggesting that MG132 treatment can increase ubiquitinated p73 levels (Figure 2B). Similar results were observed in Mdm2^−/−^mouse embryo fibroblasts (MEFs) expressing HA-tagged ubiquitin (Figure 2C). p73 (or proteins that coimmunoprecipitation with p73) appeared heavily ubiquitinated when Mdm2 was reintroduced into Mdm2 null MEFs (Figure 2C, lane 3, upper image) and the levels of p73 proteins were lower compared to that of wild type MEFs or Mdm2 null MEFs (Figure 2C, lower image). The p73 immunoblot revealed that mono- or multiple-ubiquitinated p73 occurred to a lesser extent in MDM2-siRNA than in control-siRNA cell lines, indicating an inverse correlation between the poly-and the mono-form of p73 (Figure 2A–2C, lower image). The levels of p73 did not change significantly when HEK293 cells were treated with MDM2-siRNA (Figure 2A, 2B), consistent with previous reports [18, 19]. These data indicate that MDM2 is required for p73 ubiquitination *in vivo*. To determine whether endogenous p73 and MDM2 could interact under more physiological conditions in the absence of overexpression, an IP/Western blotting experiment was performed using extracts prepared from human HEK293 cells. We observed that (i) p73 was present in the anti-MDM2 immunoprecipitates, but not in the control mouse IgG; and (ii) MDM2 was present in the anti-p73 immunoprecipitates, but not in the control mouse IgG; indicating that p73 and MDM2 physically interacts *in vivo* (Figure 2D).

### MDM2 promotes p73 ubiquitination *in vitro*

**Figure 3:**
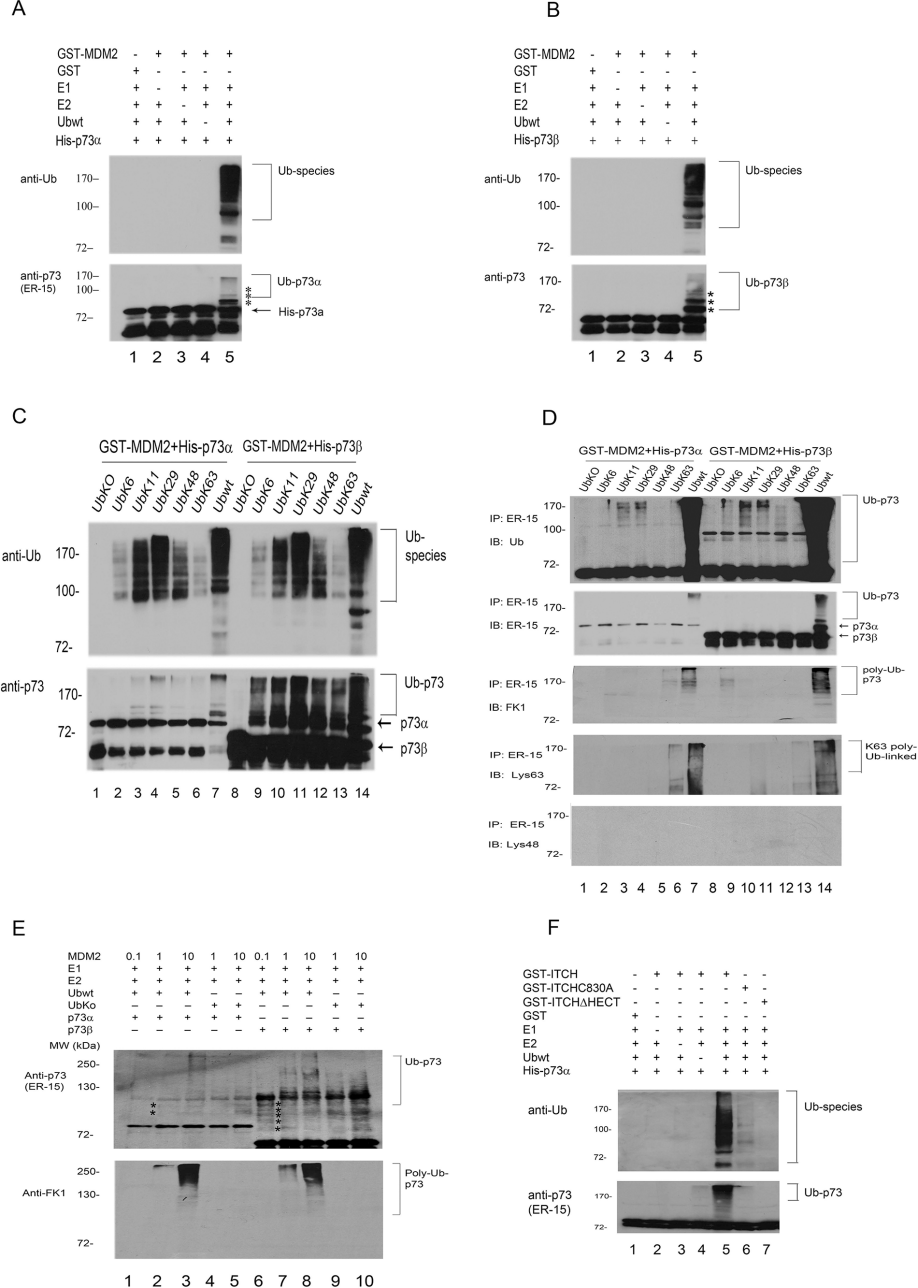
MDM2 is an E3 ligase for p73 *in vitro* **A.** and **B.** GST-MDM2 was evaluated for its capacity to ubiquitinate purified His-p73α and His-p73β using immunoblotting with anti-Ub to reveal ubiquitinated products (upper image) and an antibody directed to p73 (ER-15) to reveal ubiquitinated p73 species. **C.** MDM2 preferentially utilizes Ub with Lys residues at Lys11 and Lys29 for p73 ubiquitination *in vitro*. Affinity-purified GST-MDM2, His-p73α, or His-p73β was added to bacterial extracts containing recombinant E1 and E2 (UbcH5b), Ubwt or ubiquitin mutants. Immunoblotting was performed with anti-Ub to reveal ubiquitinated products (upper image) and anti-p73 (ER-15) to reveal ubiquitinated p73 species (lower image). **D.** As in (C) but after the ubiquitination reaction, the samples were immunoprecipitated with RIPA buffer and anti-p73 (ER-15) antibody and analyzed by immunoblotting with anti-Ub (upper image), anti-p73 (ER-15, middle image), polyubiquitin-specific FK-1 (third image), and anti-ubiquitin, Lys63-specific and Lys48-specific antibodies (lower image) as indicated. **E.** Western blot analysis of p73α or p73β ubiquitination was performed with a p73-specific antibody (ER-15, upper image), a polyubiquitinspecific antibody FK-1(lower image), and varying amounts of GST-MDM2 (0.1X: 0.3 ng; 1X: 3 ng; 10X: 30 ng) in the presence of Ubwt or UbKO. E1 and E2 were included in all reactions. Asterisk (*) indicates the migration position of p73-Ub conjugates. **F.** GST-ITCH, GST-ITCHC830A and GST-ITCHΔHECT were evaluated for E3 activity in the presence of recombinant E1, E2 (UbcH5b), ubiquitin and p73α protein. Following the ubiquitination reaction, the samples were analyzed by western blotting with ubiquitin-specific and p73-specific (ER-15) antibodies.

The experiments described above were performed in living cells, which contain endogenous E1, E2, E3, and E4 enzymes. To determine if p73 could serve as a substrate for MDM2-mediated ubiquitination *in vitro*, purified His-p73α or His-p73β was incubated with GST-MDM2 and Ubwt. As shown in Figure 3A and 3B, MDM2 promotes p73 ubiquitination dependently of the presence of E1, E2, and Ub. To further evaluate above results, a series of ubiquitin mutants that contain one lysine with the remaining six lysine residues mutated to arginine (K6, K11, K29, K48, K63) were generated, purified from *Escherichia coli,* and used for *in vitro* ubiquitination assay. In the presence of MDM2 and p73, we detected high levels of Ub-Lys11, Lys29, wt-Ub conjugation, and moderate levels of Ub-Lys6, 48, 63 conjugation (Figure 3C, upper image). The p73 immunoblots reveal that there are different patterns of ubiquitination between p73α and p73β, suggesting that MDM2 may utilize Lys11 and Lys29 of Ub to promote the ubiquitination of p73α; by contrast, MDM2 may utilize multiple residues of Ub to mediate p73β ubiquitination *in vitro* (Figure 3C, lower image). To eliminate possible autoubiquitination of MDM2, we performed coupled *in vitro* ubiquitination/IP. After a 1-hr reaction, the mixtures were immunoprecipitated with a p73-specific antibody (ER-15) and analyzed by immunoblotting with an anti-Ub monoclonal antibody to detect ubiquitinated p73 (Figure 3D, upper image), ER-15 to detect total p73 (Figure 3D, middle image), FK-1 to detect polyubiquitination of p73 (Figure 3D, third image), and anti-ubiquitin, Lys63-specific and Lys48-specific antibodies to detect Lys63 or Lys48-linked polyubiquitination of p73 (Figure 3D, lower image). Notably, p73 is polyubiquitinated by MDM2 in the presence of Ubwt and to a lesser extent in the presence of Lys63-linked chains for p73α or p73β; but not recognize by the Lys48-specific antibody (Figure 3D, lower image). These data indicate that MDM2 mainly utilizes Lys11, Lys29 and Lys63 to mediate p73 ubiquitination *in vitro*. It has been reported that low level of MDM2 induces monoubiquitination of p53, whereas high level of MDM2 promotes polyubiquitination of p53 [25]. We investigated whether p73, the structural and functional homologue of p53, can be regulated by different levels of MDM2 *in vitro*. As shown in Figure 3E (upper image), polyubiquitination of p73 was detected in the presence of high level of MDM2 with Ubwt but not UbKO. When analyzed with FK1 (Figure 3E, lower image), polyubiquitination of p73 was detected. These data suggest that like p53, p73 polyubiquitination mediated by MDM2 is dependently of the level of MDM2. Consistently, we observed that the ITCH-C830A has weak E3 ligase activity compared to that of ITCHΔHECT *in vitro* (Figure 3F). There are several cysteine residues in the HECT domain, it is possible that under certain circumstances they can serve as ubiquitin acceptors. An important consideration is why MDM2 is unable to promote p73 degradation in HEK293 cells. A recent study suggested that proteasomal degradation of some proteins requires 2 binding interactions, including polyubiquitin chains and an intrinsic proteasomal binding element in the substrates [26]. However, that study did not identify the proteasomal binding element. It is possible that the intrinsic binding element in p73 is inactive or unable to bind to the 26S proteasome. It is also possible that polyubiquitination of p73 by MDM2 primarily utilizes Lys11 or Lys29 of ubiquitin but not Lys48 (Figure 3D, lower image). Therefore, the mechanism by which MDM2 mediates p73 polyubiquitination without affecting its stability need to be further investigated.

### Overexpression of Mdm2 promotes p73 degradation in Mdm2-null MEFs

**Figure 4:**
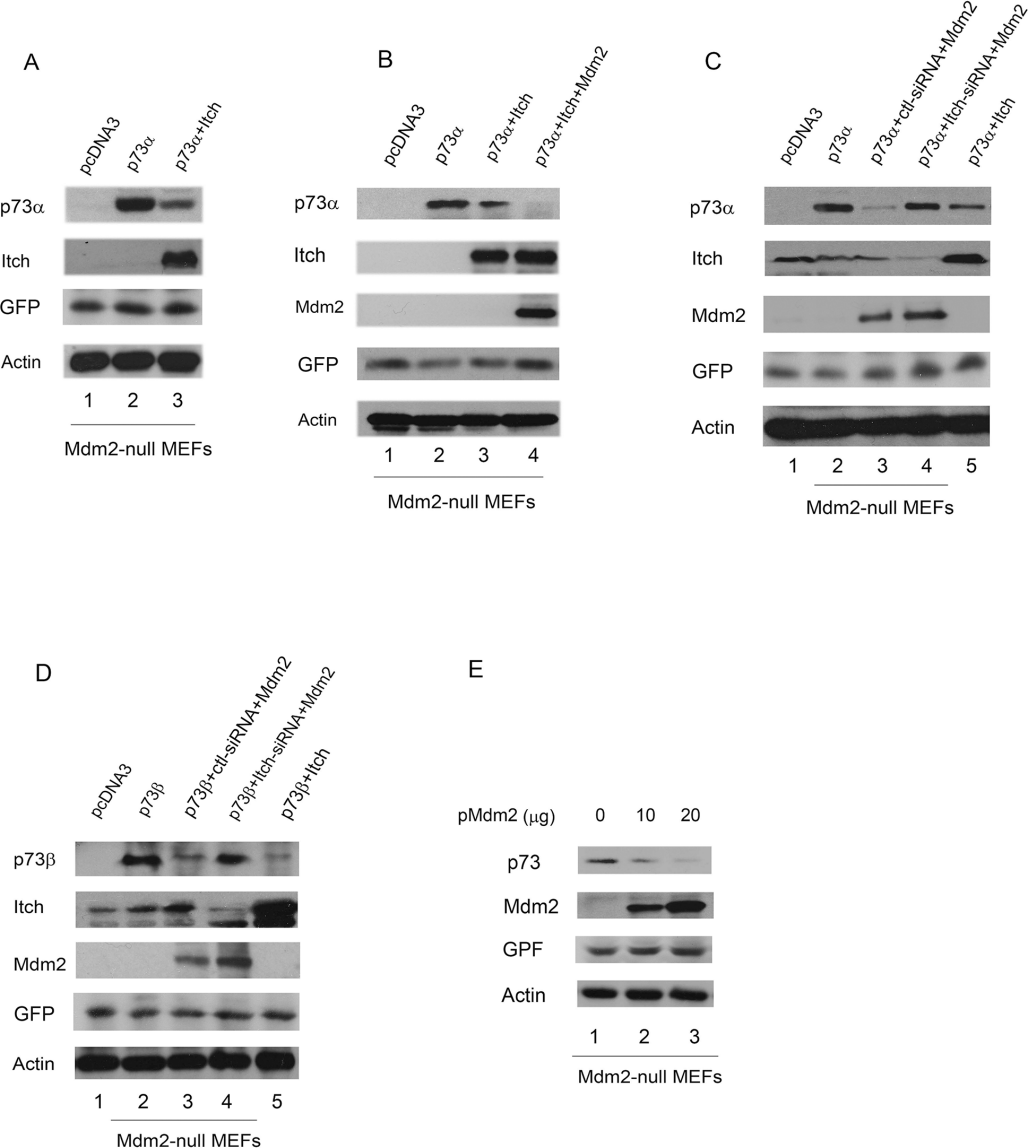
Overexpression of Mdm2 promotes p73 degradation in Mdm2-null MEFs **A.** Mdm2 null MEFs were transfected with plasmids expressing p73α or an empty vector with Myc-tagged Itch, along with a GFP expressing plasmid (pEGFP) and analyzed by western blotting with a p73-specific antibody (H-79), a Myc-specific antibody for Myc-Itch, a GFP-specific antibody (B-2), and actin as a loading control. **B.** The same procedure as (A) was used, except that Mdm2 null MEFs were cotransfected with plasmids expressing GFP, p73α along with Itch and Mdm2. **C.** and **D.** Mdm2-null MEFs were transfected with an Itch-specific siRNA or a control siRNA. Thirty hours later, the cells were transfected with plasmids expressing GFP, Mdm2 and analyzed by western blot using anti-p73, anti-Itch, anti-p53 (Pab421), anti-GFP (B-2), and anti-Mdm2 (MD-219) antibodies as indicated. **E.** Mdm2-null MEFs were transfected with plasmids expressing GFP and increased amounts of Mdm2 and analyzed by western blotting with p73-specific (H-79), GFP-specific (B-2) and Mdm2-specific (MD-219) antibodies.

Itch binds to p73 and promotes p73 ubiquitination and degradation [9]. We therefore examined whether Itch mediates p73 ubiquitination through Mdm2. Mdm2^−/−^p53^−/−^double null MEFs were transfected with plasmids expressing p73α alone or with Itch. A plasmid expressing the enhanced GFP (pEGFP) was used for measuring transfection efficiency. Extracts were prepared and analyzed by western blotting with a p73-specific antibody (H-79) and a Myc-specific antibody for Itch. As shown in Figure 4A, overexpression of Itch decreased the levels of p73 in Mdm2 null MEFs. To clarify the relationship between Itch and Mdm2, Mdm2 null MEFs were coexpressed with plasmids expressing p73α alone or with Itch or both Itch and Mdm2, and analyzed by western blotting. Coexpression of Itch and Mdm2 further increased the degradation of p73α (Figure 4B), suggesting that Mdm2 enhances Itch-mediated p73 degradation. To investigate whether Itch is required for the Mdm2-mediated degradation of p73 *in vivo*, Mdm2-null MEFs were transfected with Itch-siRNA or control siRNA. Two days later, these cells were further transfected with the Mdm2 expression plasmid and subsequently analyzed by western blot. The overexpression of Mdm2 slightly affects p73 protein levels when Itch production in these cells was eliminated using siRNA (Figure 4C, 4D). This suggests that Itch is essential for Mdm2-mediated p73 degradation in Mdm2-null MEFs. To determine whether overexpression of Mdm2 regulates the level of endogenous p73 protein, Mdm2-null cells were transfected with increasing amounts of Mdm2 expression plasmid. The transient overexpression of Mdm2 decreased the levels of endogenous p73 protein (Figure 4E), suggesting that the p73 proteins are target of Mdm2.

### Mdm2 interacts with Itch and regulates the stability of p73 proteins

**Figure 5:**
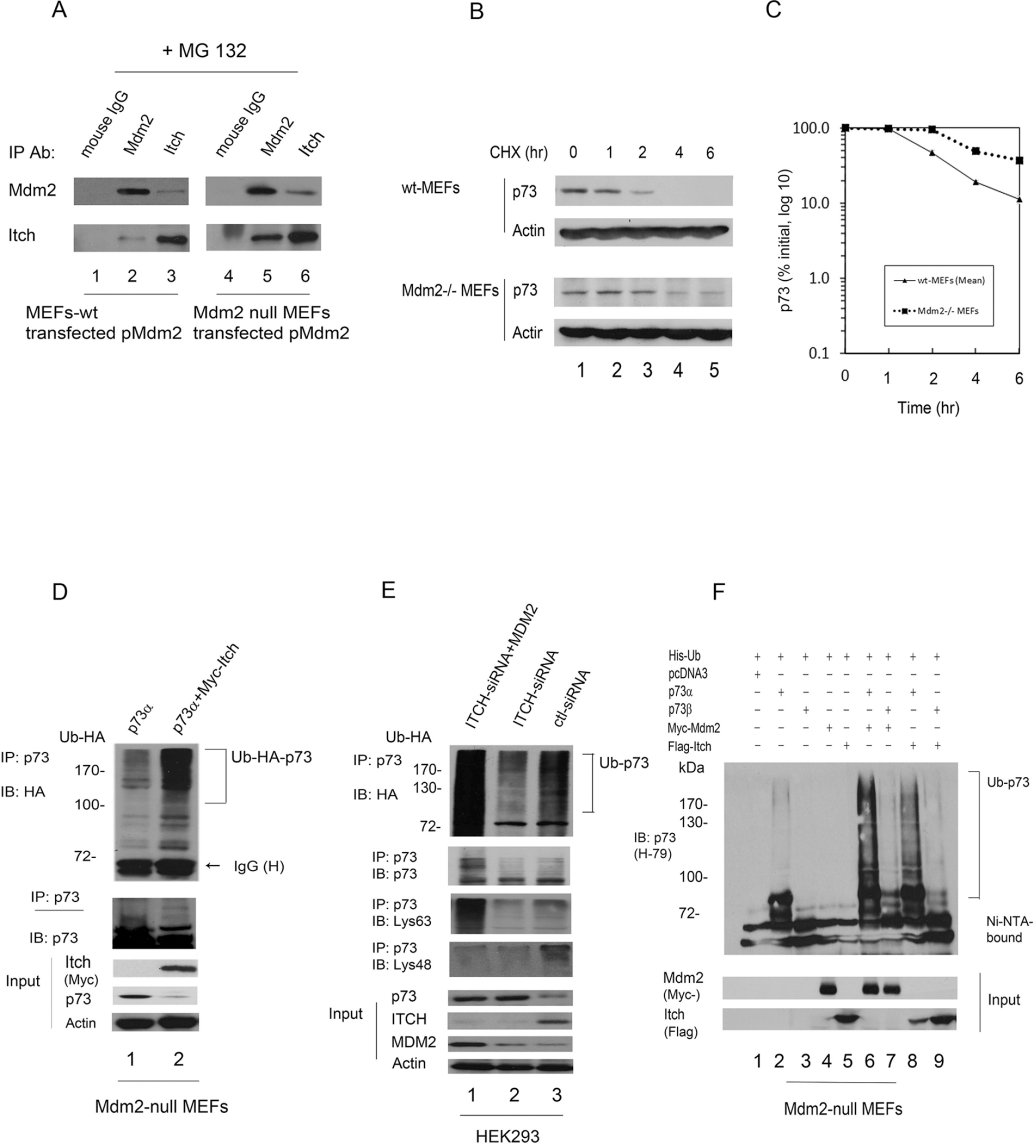
Mdm2 interacts with Itch and regulates the stability of p73 in Mdm2-null MEFs **A.** Mdm2 interacts with Itch *in vivo*. Wild type MEFs and Mdm2-null MEFs were transfected with a plasmid expressing Mdm2. The cells were treated with the proteasome inhibitor MG132 (20 μM) for 6 hours prior to harvest. The cell extracts were immunoprecipitated with anti-Mdm2 or anti-Itch antibodies and analyzed by western blot with indicated antibodies. **B.** Wild type MEFs and Mdm2 null MEFs cells were treated with cycloheximide (CHX) (20 μg/ml) as indicated. Endogenous p73 levels were determined by immunoblotting with a p73-specific antibody (H-79). An antibody against β-actin was used as a loading control. **C.** Expression levels were determined by densitometry of the immunoblots in (B) Errors bars indicate the SEM (*n* = 3). **D.** Mdm2 null MEFs were transfected with plasmids expressing HA-Ub and p73α or Myc-Itch. Cell extracts were immunoprecipitated with a p73-specific antibody (H-79) and analyzed by western blotting with HA-specific, Myc-specific (for Itch), and p73-specific antibodies. **E.** HEK293 cells were transfected with ITCH-siRNA or control-siRNA. Two days later, the cells were further transfected with plasmids expressing HA-Ub and an MDM2 expression plasmid or empty vector as indicated. Cell extracts were immunoprecipitated with a p73-specific antibody (ER-15) and analyzed by western blotting with HA-specific, p73-specific, Lys63-specific and Lys48-specific antibodies (lower image) as indicated. Direct western blots for p73, ITCH, MDM2 and actin are shown in the lower panel. **F.** Mdm2 null MEFs were transfected with plasmids expressing His-Ub, p73α or p73β, and Mdm2 or Itch. His-ubiquitinated proteins were isolated from denatured whole extract extracts, and analyzed by western blot with a p73 specific antibody (H-79). Direct western blots for Mdm2 and Itch are shown in the lower panels.

MDM2 was recently reported to promote p73 degradation through interaction with ITCH in HeLa cells [27]. We were able to detect weak interaction between Itch and transfected Mdm2 in wild type MEFs compared to that of Mdm2 null MEFs in the presence of the proteasome inhibitor MG132 (Figure 5A). To further determine if Mdm2 regulates p73 stability, wild type MEFs and Mdm2 null MEFs were treated with cycloheximide to inhibit *de novo* protein synthesis. We observed that the half-life of endogenous p73 was approximately 1 hr in wild type MEFs. By contrast, the half-life of p73 increased to approximately 3 hr in Mdm2 null MEFs (Figure 5B, 5C). These data reveal that Mdm2 is able to regulate the stability of p73 in Mdm2 null MEFs. In the co-IP experiment, immunoprecipitated p73 was heavily ubiquitinated in the presence of Itch (Figure 5D). The data indicates that Itch functions independently of Mdm2. Furthermore, we investigated whether MDM2 mediates p73 ubiquitination through ITCH in different cell types. Endogenous ITCH was subjected to ablation by ITCH-siRNA in human HEK293 cells. Two days later, cells were transfected with MDM2 and HA-tagged ubiquitin (HA-Ub) expression plasmids. Extracts were immunoprecipitated with a p73-specific antibody (ER-15) and analyzed by western blotting with an HA-specific antibody to detect ubiquitinated p73 (Figure 5E, upper image) and a p73-specific antibody (ER-15) to detect all p73 (Figure 5E, lower image). Heavily ubiquitinated p73 (or proteins that coimmunoprecipitated with p73) was detected when the ITCH-siRNA treated cells transfected with MDM2 compared with cells transfected with an empty vector or control-siRNA (Figure 5E). The p73 immunoblot also reveal ubiquitination of p73 when cells were transfected with MDM2; however, the levels of p73 were not significantly changed in HEK293 cells (Figure 5E, lower image). These data indicate that MDM2 promotes p73 ubiquitination independently of ITCH. We further tested the ability of MDM2 to regulate the level of p73 in HEK293 cells. Notably, the half-life of p73 remained unchanged in the presence of MDM2 compared with pcDNA3 (Supplementary Figure S2A). By contrast, the half-life of p73 was greatly decreased in the presence of ITCH. We also observed that the half-life of endogenous p73 in the presence of control-siRNA or MDM2-siRNA was approximately 4 hr. The half-life increased to approximately 6 hr in cells depleted of ITCH (Supplementary Figure S2B). These data revealed that MDM2 is unable to regulate the stability of p73 in HEK293 cells. Moreover, we confirmed that p73α was heavily ubiquitinated in the presence of Mdm2 or Itch and p73β was a lesser extent in the presence of Mdm2 or Itch in Mdm2 null MEFs using His ubiquitin pull down assay (Figure 5F).

### MDM2 represses p73 apoptosis and cell cycle arrest but not p73-dependent transcriptional activity via E3 ligase activity in tested cells

**Figure 6:**
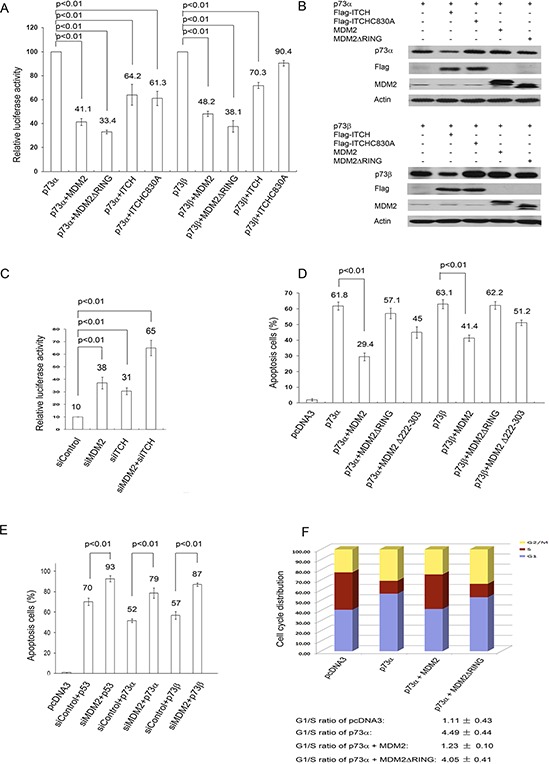
MDM2 inhibits p73-dependent apoptosis and cell cycle arrest but not p73-dependent transcriptional activity **A.** H1299 cells were cotransfected with a p21-Luc reporter plasmid and the p73α or p73β expression construct in combination with the MDM2, MDM2ΔRING, ITCH, or ITCH C830A expression constructs or empty vector (pcDNA3.1). The transcriptional activity of p73 is shown, and error bars indicate the SEM (*n* = 3). *p* < 0.01 (2-tailed Student *t* test). **B.** Western blots of p73α, p73β, ITCH, ITCHC830A, MDM2 and MDM2ΔRING with p73-specific (ER15), Flag-specific for ITCH and ITCHC830A and MDM2-specific antibodies were indicated. **C.** HEK293 cells were transfected with MDM2-siRNA (siMDM2), ITCH-siRNA (siITCH), control-siRNA, or MDM2-siRNA and ITCH-siRNA. Two day later, the cells were transfected with a p21 Luc reporter plasmid and a p73α expression plasmid. The transcriptional activity of p73 is shown, and error bars indicate the SEM (*n* = 3). *p* < 0.01 (2-tailed Student *t* test). **D.** H1299 cells were cotransfected with a CD20 expression construct, pcDNA3-p73α, or p73β (3 μg) and pcDNA3-MDM2 (15 μg), pcDNA3-MDM2ΔRING (15 μg), or pcDNA3-MDM2Δ222–303 as indicated. The inhibitory effect of MDM2 on p73-dependent apoptosis was determined by annexin V staining of CD20-positive cells and flow cytometry. Error bars indicate the SEM. (*n* = 3). *p* < 0.01 (2-tailed Student *t* test). **E.** H1299 cells were transfected with MDM2-siRNA or control-siRNA for 30 h. The cells were then transfected with the p53, p73α, or p73β expression constructs and CD20 expression plasmids. The number of surviving CD20-positive cells was measured by flow cytometry 24 hr after transfection. *p* < 0.01 (2-tailed Student *t* test). **F.** Human bladder carcinoma EJ cells were transfected with p73α alone or with MDM2, MDM2ΔRING, or empty vector (pcDNA3). The cell cycle profile was determined by propidium iodide staining and flow cytometry. The results represent the average of triplicate experiments.

To determine the functional consequences of the interaction of MDM2 with p73, we examined the effect of MDM2 or ITCH expression on p73mediated transcriptional activity. H1299 cells were cotransfected with a luciferase reporter construct (p21-luc) containing the p53 binding site from the p21^WAF1^ promoter and p73 alone or with MDM2 or ITCH. Both MDM2 and ITCH repress p73mediated transactivation activity (Figure 6A), consistent with previous studies [9, 18, 19]. Notably, the MDM2ΔRING mutant significantly repressed p73-dependent transactivation, suggesting that MDM2 impairs the transactivation function of p73 without targeting it for degradation and indicating that the RING domain of MDM2 is not necessary for MDM2 inhibition of p73 transcriptional activity. A portion of cell extracts were lysed and analyzed by western blots (Figure 6B). To further assess the involvement of endogenous MDM2 in the regulation of p73 ubiquitination, HEK293 cells were pretreated with control-siRNA, MDM2-siRNA, ITCH-siRNA, or MDM2-siRNA and ITCH-siRNA. The cells were also transfected with the p21^WAF^-luc reporter. As shown in Figure 6C, the activity of p21^WAF^-luc was increased in cells depleted of MDM2 or ITCH by siRNA. The transcriptional activity of p73 was further increased in cells depleted of both MDM2 and ITCH (Figure 6C). This is consistent with the observation that p73 transcriptional activity is inhibited by MDM2 overexpression (Figure 6A). The mechanism of MDM2-mediated inhibition of p73 transactivation activity involves MDM2 binding to the N-terminal domain of p73, which blocks the ability of p73 to activate target gene transcription. Next, we examined whether overexpression of MDM2 inhibits p73-dependent apoptosis. Transient expression experiments followed by FACS analysis (using annexin V staining) were performed to determine whether MDM2 expression can rescue cells from p73-dependent cell death. As shown in Figure 6D, expression of p73 alone resulted in apoptosis. However, apoptosis is largely prevented by coexpression of MDM2 [19]. The MDM2ΔRING mutant was unable to rescue cells from p73-dependent cell death, which suggests that the E3 ligase activity of MDM2 is required to inhibit p73-mediated apoptosis. The acidic domain (AD) of MDM2 has been reported to play a critical role in p53 ubiquitination [28, 29]. Neither the RING nor the AD domain of MDM2 alone is sufficient for efficient p53 ubiquitination [28, 29]. However, we observed that the MDM2Δ222–303 mutant (deletion of AD of MDM2) was able to partially prevent p73-dependent apoptosis, indicating that the AD domain of MDM2 is not absolutely necessary for the repression of p73dependent apoptosis (Figure 6D). This indicates that MDM2-mediated p73 ubiquitination is linked to inhibition of the apoptotic function of p73. To further determine whether endogenous MDM2 is required to inhibit p73-dependent apoptosis, H1299 cells were exposed to either control-siRNA or MDM2-siRNA. Two days later, these cells were transfected with p53, p73α, or p73β and analyzed by flow cytometry using annexin V staining. Our findings revealed that depletion of MDM2 resulted in additional apoptosis when cells were transfected with p53, p73α, or p73β, confirming that MDM2 plays an important role in regulating p73 apoptosis (Figure 6E).

Cell cycle arrest mediated by p73 is an important tumor suppression function of p73. To determine whether MDM2 can inhibit p73-induced G1 arrest, human bladder carcinoma EJ cells, which lack functional p53 [30, 31], were transfected with a plasmid expressing p73α alone, with MDM2, or with MDM2ΔRING. Cell cycle analysis by propidium iodide staining and flow cytometry was performed. The relative proportion of cells in each phase (G1, S and G2/M) of the cell cycle was determined using the automated ModFit program (Verity Software House Incorporated). It is important to compare the G1/S ratios; an increase in the G1/S ratio has been used as an indicator of G1 arrest [20–22]. Overexpression of p73α exhibited an increase in the proportion of cells in G1 and a decrease in the proportion of cells in S phase, resulting in an increase in the G1/S ratio from 1.11 to 4.49 (Figure 6F). We observed that p73-induced G1 arrest was partially prevented by overexpression of MDM2; however, MDM2ΔRING was unable to prevent p73-induced G1 arrest (Figure 6F), suggesting that the E3 ligase activity of MDM2 is required to inhibit p73-dependent cell cycle arrest. These data demonstrate that the E3 ligase activity of MDM2 is involved in the regulation of p73-dependent apoptosis and checkpoint control.

## DISCUSSION

The protein p73 was isolated as a p53 homolog and exhibits similar functions, such as transactivation of p53 target genes, induction of apoptosis, and growth suppression. MDM2 has been reported to bind to p73 and inhibits p73dependent transactivation and apoptosis. While MDM2 regulates p53 stability, it is not involved in the regulation of p73 protein stability [18, 19]. It has been reported that Nutlin-3, a small molecule inhibitor of MDM2, can disrupt endogenous p73-MDM2 binding and induce apoptosis in the absence of p53 [32]. Busuttil et al observed that NF-κB induced Mdm2 expression in activated T cells [33]. Mdm2 prevents Bim-mediated apoptosis via binds and inhibition of p73 [33]. However, the molecular mechanism by which MDM2 represses p73 function is not clear. In this study, we elucidated the mechanism by which MDM2 negatively regulates p73 function. This is the first report in which p73 ubiquitination mediated by MDM2 has been linked to MDM2 repression of p73-dependent apoptosis and cell cycle arrest but not for p73-dependent transcriptional activity.

To investigate the role of MDM2 in a physiological setting, endogenous MDM2 was depleted by siRNA. We demonstrated that p73 ubiquitination was greatly decreased in cells depleted of MDM2, suggesting that MDM2 is required for p73 ubiquitination *in vivo*. We also observed that overexpression of MDM2 promoted p73 ubiquitination. By contrast, MDM2ΔRING was unable to mediate p73 ubiquitination, indicating that the E3 ligase activity of MDM2 is required for p73 ubiquitination. Intriguingly, MDM2 utilizes multiple Ub chains (Lys11, Lys29, and Lys63) to mediate p73 ubiquitination *in vivo* and *in vitro*. It has been reported that Lys11-, Lys 29-, and Lys48 linked chains are targeted for degradation by the 26S proteasome [34, 35]. Lys63-linked chains appear to be used primarily for nonproteasomedependent regulation of processes such as DNA repair, endocytosis, and chromatin remodeling [7, 36]. The biological importance of K48-linked polyubiquitinated chains is confirmed by the lethality of the K48R mutation in the yeast *Saccharomyces cerevisiae* [37]. Monoubiquitination is involved in a variety of cellular functions, including transcriptional activation, protein-protein interactions, and membrane trafficking [38]. Our findings revealed that high level of MDM2 mediates polyubiquitination of p73. We detected p73 polyubiquitination in the presence of Ubwt and weak p73 polyubiquitination in the presence of Lys63 or Lys6 chains. This may explain why MDM2 is unable to degrade p73 proteins in certain cell types. A recent study proposed that degradation of some polyubiquitinated proteins requires two binding interactions, including polyubiquitin chains and an intrinsic proteasomal binding element in the substrates [26]. It is also possible that the proteasomal binding element in p73 is inactive. Additional studies are required to address this molecular mechanism.

MDM2 did not promote p73 degradation in most tested cell lines. Recently, Kubo et al. reported that MDM2 mediates p73 degradation through interaction with Itch in HeLa cells [27]. Our finding that MDM2 has an undetectable effect on p73 at the protein level in certain cell types is in agreement with previous studies. We were unable to detect an MDM2 interaction with ITCH in H1299 and HEK293 cells, also consistent with previous reports [27]. We show that Itch (the mouse homolog of ITCH) degrades p73 independently of Mdm2 in Mdm2 null MEFs. Notably, overexpression of Mdm2 promotes p73 degradation in Mdm2-null MEFs. We demonstrate that Itch is essential for overexpression of Mdm2 mediated p73 degradation in Mdm2-null MEFs. Moreover, we observe that the E3 ligase activity of MDM2 is required for MDM2 to suppress p73-dependent apoptosis and cell cycle arrest. Our findings provide evidence for a link between the E3 ligase activity of MDM2 and its role in repressing p73 function.

An important question raised by our results is whether the stability of p73 is regulated by MDM2 through distinct mechanisms. First, MDM2 interacts with ITCH in certain cell types, which suggests that additional co-factor(s) are required for the interaction. No evidence showed that MDM2 directly bound to ITCH *in vitro*. Second, the complex formation between MDM2 and ITCH may not be stable in some cell types. That is partially explained why MDM2 repressed p73-dependent apoptosis in some cell types, which we do not see significant p73 degradation. Third, it is possible that additional co-factor(s) inhibit the interaction between MDM2 and ITCH in some cell types. However, this inhibition is removed/or turned off in certain cell types (such as HeLa and Mdm2-null MEFs). Mdm2 and Itch are E3 ubiquitin ligases that regulate p73 ubiquitination and degradation. Both the RING domain and the HECT domain share similar surface structures in E2 binding, but use distinct mechanisms for substrate ubiquitination. It will be interesting to further address the effect of Mdm2 on Itch mediated p73 regulation and function.

Controlling p73 stability is critical to understanding the regulation of the protein activity in the cell. In this study, we elucidated the molecular mechanism by which MDM2 inhibits p73 function. Our findings also demonstrated that MDM2 is a critical negative regulator of p73.

## MATERIALS AND METHODS

### Plasmids and antibodies

GST-MDM2, p21-Luc, pCMV-Bam-MDM2, and MDM2ΔRING have been described previously [20, 21]. Myc-Mdm2 was kindly provided by Dr. Jochemsen. All Ub and Ub mutants were PCR-amplified and subcloned into pET28a. The mouse Mdm2 was also cloned into pcDNA3 and confirmed by sequence. Flag-p73α, p73β, and p73 mutants were generated by PCR and subcloned into pCMV-Tag1 (Stratagene). p73α and p73β were also cloned into pcDNA3 without tag. All PCR products were confirmed by sequencing. Anti-p73 (Ab-1 and Ab3, Oncogene Science; H-79, Santa Cruz Biotechnology; ab40658, Abcam), anti-MDM2 (2A10, Calbiochem; SMP14, Santa Cruz Biotechnology; MD-219, Sigma), anti-Myc (9E10, Roche), anti-Flag (M5, Sigma), anti-GFP (B-2, Santa Cruz Biotechnology), anti-GST (B-14, Santa Cruz Biotechnology), anti-HA (12CA5, Roche), anti-ubiquitin (BD Bioscience), anti-actin (Sigma), anti-CD20 (Pharmingen), and polyubiquitin-specific FK-1, anti-ubiquitin, Lys63-specific and Lys48-specific antibodies (Millipore) were used according to the manufacturers’ instructions.

### Cell culture and DNA transfection

All cells were maintained in Dulbecco's modified Eagle's medium (DMEM) supplemented with 10% fetal bovine serum. The p53^−/−^Mdm2^−/−^double null MEFs were kindly provided by Dr. G. Lozano. H1299 cells and Saos-2 cells were transfected using the calcium phosphate method. Mdm2 null MEFs were transfected using Lipofectamine 2000 (Invitrogen).

### siRNA experiments

For siRNA experiments, HEK293 cells were transfected with siRNA using Lipofectamine 2000 (Invitrogen). The Itch target sequences were AAGTGCTTCTCAGAATGATGA and AACCACAA CACACGAATTACA, and the scrambled sequence was AATTCTCCGAACGTGTCACGT [as described previously (9)]. pSuper.neo.gfp-Mdm2 siRNA (GACAAAGAAGAGAGTGTGG) was a kind gift from Dr. C. Blattner.

### Expression and recombinant protein preparation

All GST or His-tagged recombinant proteins were expressed in *E. coli* BL21 (DE3) (Novagen), treated with isopropyl-β-D-thiogalactoside to induce fusion protein expression, and purified using glutathione Sepharose 4B (Amersham) for GST-fusion proteins or Ni^2^-NTA agarose (Qiagen) for His-fusion proteins.

### Immunoprecipitation and measurement of p73 half-life

Cells were lysed in 50 mM Tris-HCl (pH 8.0), 5 mM EDTA, 150 mM NaCl, 0.5% NP-40 containing a protease inhibitor tablet (Roche) and immunoprecipitated with specific antibodies. The immune complexes were collected with protein A agarose beads and washed 4 times with lysis buffer. The immunoprecipitates were analyzed by SDS PAGE followed by autoradiography. To measure the p73 half-life, wild type MEFs and Mdm2 null MEFs were treated with 25 μg/ml cycloheximide (CHX) to inhibit *de novo* protein synthesis. Protein levels were monitored by immunoblotting with a p73 specific antibody (H-79) at the indicated time points. The relative amount of p73 protein was determined by densitometry and normalization using β-actin.

### *In vitro* ubiquitination assay

The *in vitro* ubiquitination assay was performed as described previously [20–22] with some modifications. For MDM2-mediated ubiquitination, rabbit E1 (20–40 ng, Calbiochem), UbcH5b (100 ng, Calbiochem), ubiquitin or His-ubiquitin (5 μg, Sigma), His-p73, and GST-MDM2 (0.2–0.5 μg) were added to ubiquitination buffer (50 mM Tris-HCl, pH 7.4, 2 mM ATP, 5 mM MgCl2, and 2 mM DTT) to obtain a final volume of 30 μl. The reactions were incubated at 30°C for 1.0–1.5 hr. The reactions were stopped with 2x SDS loading buffer, resolved on SDS-PAGE gels, and analyzed by western blotting.

### *In vivo* ubiquitination assay

Cells were transfected with expression plasmids encoding p73, MDM2, or Itch alone or in combination with HA-tagged ubiquitin, HA-ubiquitin mutants, Myc-Ubwt, or a Myc-Ub4KR mutant. After 30 hr, cells were harvested, lysed with RIPA buffer [50 mM Tris-HCl pH 7.6, 150mM NaCl, 1% NP-40, 1% sodium deoxycholate, 0.1% SDS, and protease inhibitors (Roche)], and immunoprecipitated with the indicated antibodies. Immune complexes recovered with protein A-Sepharose were washed 4 times with RIPA buffer, separated by 10% SDS-PAGE, and analyzed by IP/immunoblotting as described previously [20–22].

### His-ubiquitin pull-down assay

Cells were transfected with His-tagged ubiquitin and indicated expression plasmids. Thirty hours after transfection, cells were resuspended in Buffer A (6 M guanidine-HCl, 0.1 M Na2HPO4/NaH2PO4, 10 mM Tris-HCl, pH 8.0, 10 mM imidazole at pH 8.0) and sonicated. Cell lysates were added to 50 μl of equilibrated Ni-NTA agarose and were allowed to incubate for 3 h at room temperature. Beads were then washed one time with Buffer A, followed by two washes with Buffer A/TI (1 vol of Buffer A, 3 vol of Buffer TI [25 mM Tris-Cl, 20 mM imidazole at pH 6.8]), and one wash with Buffer TI; all washes were 1 ml. After extensively washing, the precipitates were boiled with SDS loading buffer and then subjected to SDS-PAGE followed by immuno-blot analysis.

### Cell cycle analysis

(described previously [20–22]).

### Apoptosis assay

(described previously [20–22]).

### Luciferase assay

As described previously [20–22], pGL3-E1bTATA contains a minimal promoter consisting of a TATA box downstream of one copy of the p53 binding site from the 5′end of the *p21WAF* promoter, referred to as p21-Luc. A β galactosidase reporter construct, pCMV-β-gal (Promega), was included in all the transfection mixtures. Luciferase activity was measured 2 days posttransfection on samples containing equivalent amounts of protein using an LB9507 luminometer and the luciferase assay reagent (Promega). All values were normalized to β-galactosidase activity.

## SUPPLEMENTAY FIGURES


