# Supplementary material for: MDM2 mediates p73 ubiquitination: a new molecular mechanism for suppression of p73 function
Source: Oncotarget. 2015 May 26;6(25):21479–92. doi: 10.18632/oncotarget.4086 (PMC4673280; doi:10.18632/oncotarget.4086)
Supplement: Supplementary file 1 [file oncotarget-06-21479-s001.pdf]

SUPPLEMENTAY FIGURES

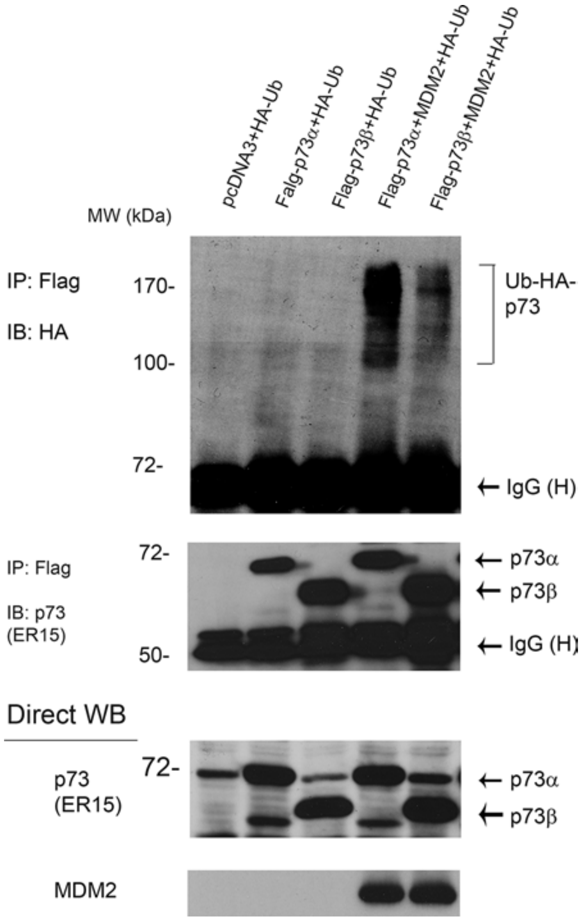

Supplementary Figure S1: MDM2 promotes p73 ubiquitination in H1299 cells. H1299 cells were transfected with plasmids expressing Flag-p73α, Flag-p73β, or in combination with pcDNA3-MDM2 and HA-tagged ubiquitin. p73 was immunoprecipitated with an anti-Flag (M5) mAb and analyzed by immunoblotting with a HA antibody (top) or with the ER-15 antibody for p73 (bottom). Direct Western blots for p73α, p73β, and MDM2 are shown in the bottom panels.

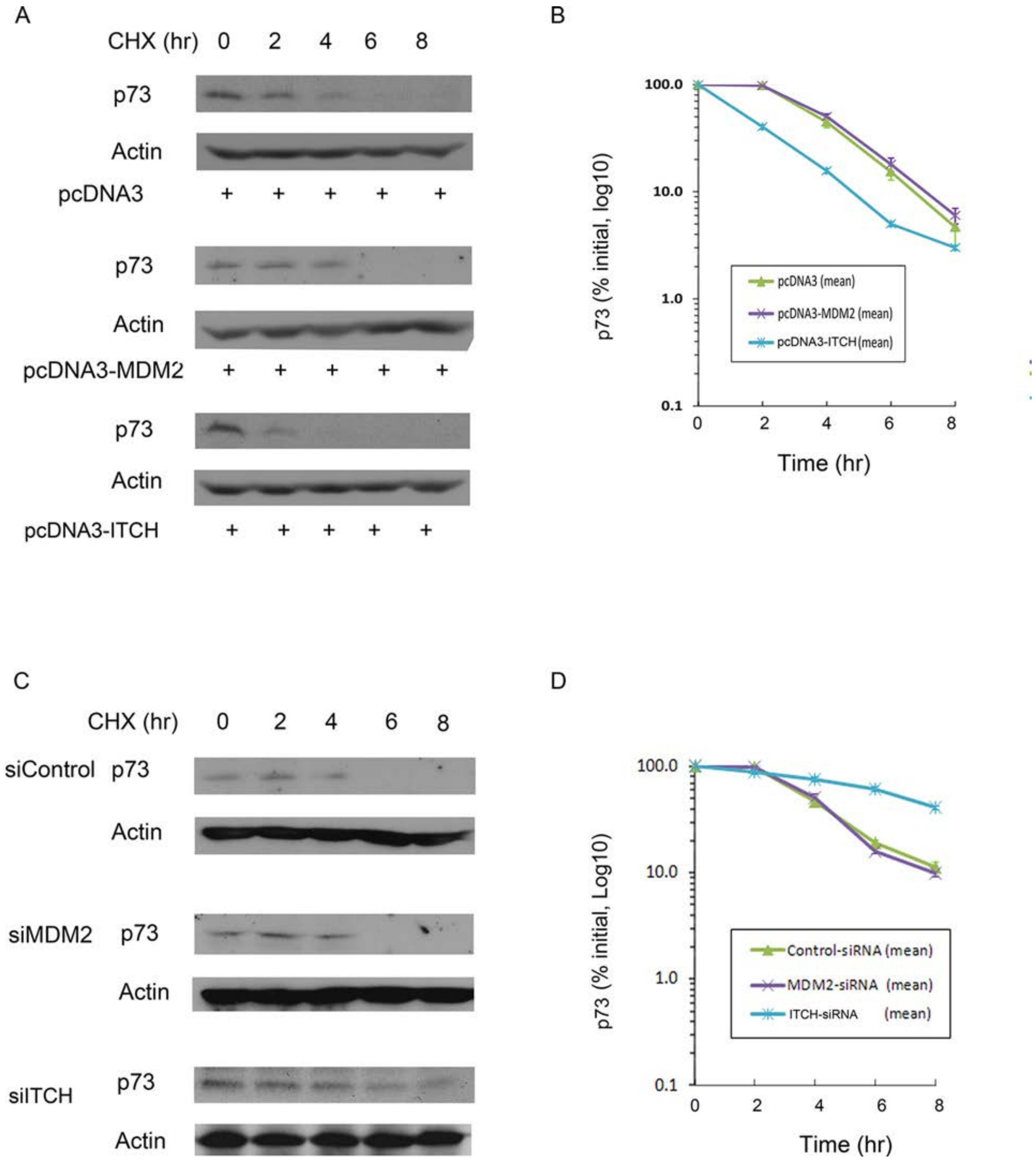

Supplementary Figure S2: MDM2 ablation by siRNA does not affect p73 half-life in HEK293 cells. A. HEK293 cells were transfected with an empty vector, an MDM2 expression plasmid, or an ITCH expression plasmid in the presence of cycloheximide (CHX) (20 µg/ml). Endogenous p73 levels were determined by immunoblotting with a p73-specific antibody (ER-15). An antibody against β-actin was used as a loading control. **B.** Expression levels were determined by densitometry of the immunoblots in (A) Errors bars indicate the SEM ($n = 3$). **C.** HEK293 cells were transfected with control-siRNA (siControl), MDM2-siRNA (siMDM2), or ITCH-siRNA (siITCH) in the presence of cycloheximide (CHX) (20 µg/ml). Endogenous p73 levels were measured by immunoblotting with a p73-specific antibody (ER-15). An antibody against β-actin was used as a loading control. **D.** Expression levels were determined by densitometric analysis of the immunoblots in **C.** Errors bars indicate the SEM ($n = 3$).
